# Morrison and Brandreth’s Pills

**Published:** 1837

**Authors:** 


### Art. V.—Morrison and Brandreth's Pills.

Mankind love the luxury of delusion in physic, as well as in politics and religion. Quackery has ever flourished side by side with true science. So it is, so it ever has been, and so it will be, till there is no further need of pills or politics or prayers.—But it is nevertheless the duty of sound hearted and better instructed men, to withstand the encroachments of ignorant presumption, and empyrical knavery. We should oppose truth and fair dealing, to low-minded cunning and mercenary artifice.

In a late number of this Journal was published, from a highly respectable foreign writer, a cutting stricture on the eulogies bestowed by some of our American medical magnates on Swaim's Panacea. We deeply regretted *the necessity* which provoked the comment alluded to, but by no means regret the *comment itself.*

England, *par excellence*, has been designated the paradise of quacks. America is not much inferior to the mother country, in the fostering smiles with which all kinds of quackery are looked upon. Besides English quack medicines, such as Morrison's pills, Bateman's cordial drops, Godfrey's cordial, &c. &c. we have domestic manufactured articles in this line, such as Sherwood's Galen pills, Gardner's Indian balsam, &c. &c.

Were these medicines possessed of a merely negative character of evil, they would deserve reprehension. For by the high confidence which they create in reference to their curative capabilities, they often injure by excluding proper medicines in proper periods of disease. But the mischief stops not on the side of negation—they inflict positive and wide-spread injury, by their active irritating powers.

Both Morrison's and Brandreth's pills, are of a drastic, irritating character. When the lower bowels are loaded with feculent materials, they may, by a slow and sure dislodgment of the accumulation, relieve. But when taken, as they often are, in fever, or when the intestinal canal is in a state of irritation, they may bring on inflammation of the mucous coat. We had a case under our care lately, which afforded a melancholy exemplification of the above remark. The patient was an old lady of good constitution, who took sixteen of Brandreth's pills in three days, in order to clear the *blood* of its bad humors; for she assured us, she was well persuaded of the truth of the averment made by Brandreth, that "impurity of blood" was the cause of her ailment. The old lady had a slight attack of vernal intermittent, and it was for this she took the pills. Great intestinal irritation arose from their activity, and erysipelas came out on her nose and face. This she was glad to see, as the humors were coming out—were driven to the exterior from the interior, by the great power of the said pills. When called to see her, two days after the erysipelatous inflammation commenced, and four days after taking the last dose of pills, we found her with fever, foul tongue, sordid, dry secretion about the teeth, great thirst, irritable stomach, perpetual burning sensation in the præcordial region, and considerable intumescence of face, from the diffusion of the inflammation over both cheeks and eyes, and part of the scalp. The erysipelas was arrested at first by strong mercurial ointment, applied by gentle friction over the red spots, and along their margins; but it renewed its march in a few days, and although blisters were applied, it continued to spread till delirium came on, and she died. No remedy which we administered abated the intense burning sensation in the præcordial region.

In Prof. Dunglison's Medical Library, we find the outlines of the history of a trial, which occurred in London in last February. Morrison & Moat, manufacturers and venders of the "Morrison's pills," brought an action against Alderman Harmer, proprietor of the London Weekly Despatch, for a libel, the language of which was as follows:—"Since we commenced exposing the homicidal tricks of these impudent and ignorant scamps, who had the audacity to pretend to cure all diseases with one kind of pills, which pills were composed of nothing more nor less than gamboge and aloes, several of the rotgut rascals have been convicted of manslaughter, and fined and imprisoned for killing people with enormous doses of 'universal vegetable boluses.'"

Professor Daniel, of King's College, London, stated, on the trial, that he had, by analysis, found that Morrison's pills contained the following ingredients: No. 1 consisted of aloes, eleven and one-tenth grains; cream of tartar, ten and four-tenths grains; gum and soluble matter, four and four-tenths grains—in twelve pills.

No. 2 consisted of aloes, five and one-tenth grains; gamboge, four and one-tenth grains; colocynth, two grains; gum, four and seven-tenths grains; cream of tartar, six and seven-tenths.

Mr. Hume, the chemist, corroborated, by his statement, Dr. Daniel's representation of the composition of the pills.

Sir H. Halford and Dr. James Johnson were called on to depose, respecting the safety or noxious quality of the pills. They considered the pills as dangerous, when employed as they usually are in large doses, and in an indiscriminate manner.

"The jury, by their verdict, denounced the pills as dangerous; but added, that they thought they might be taken, if administered with proper care and discretion." H.

